# Supplementary material for: Reliance upon ancestral mutations is maintained in colorectal cancers that heterogeneously evolve during targeted therapies
Source: Nat Commun. 2018 Jun 12;9:2287. doi: 10.1038/s41467-018-04506-z (PMC5997733; doi:10.1038/s41467-018-04506-z)
Supplement: Supplementary file 1 — Supplementary Information [file 41467_2018_4506_MOESM1_ESM.pdf]

Reliance upon ancestral mutations is maintained in colorectal cancers that heterogeneously evolve during targeted therapies

Russo et al.

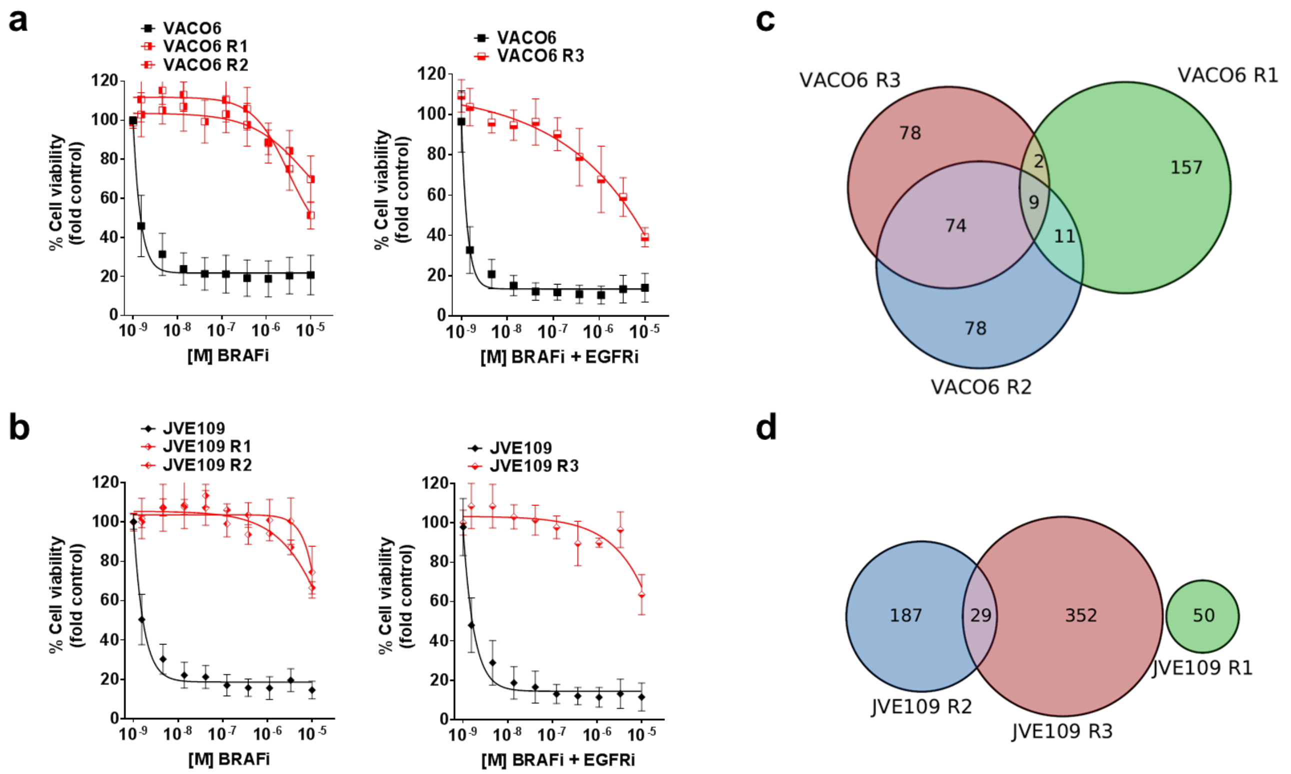

Supplementary Figure 1. *Acquisition of secondary resistance to MAPK inhibition in CRC cells.* BRAF mutated VACO6 (a) and JVE109 (b) CRC cells were treated with dabrafenib (BRAFi) alone or in combination with cetuximab (EGFRi) till resistant populations emerged. A 5-days cell viability assay was used to validate resistance. Results represent means ± SD of at least two independent experiments. (c-d) Venn diagrams show the number of somatic mutations acquired at resistance to targeted therapies in each indicated CRC population. Each area section is proportional to the drawn number. Full color indicates "private" acquired variations; while shared mutations are showed in mixed color. Numbers indicate common and shared variations.

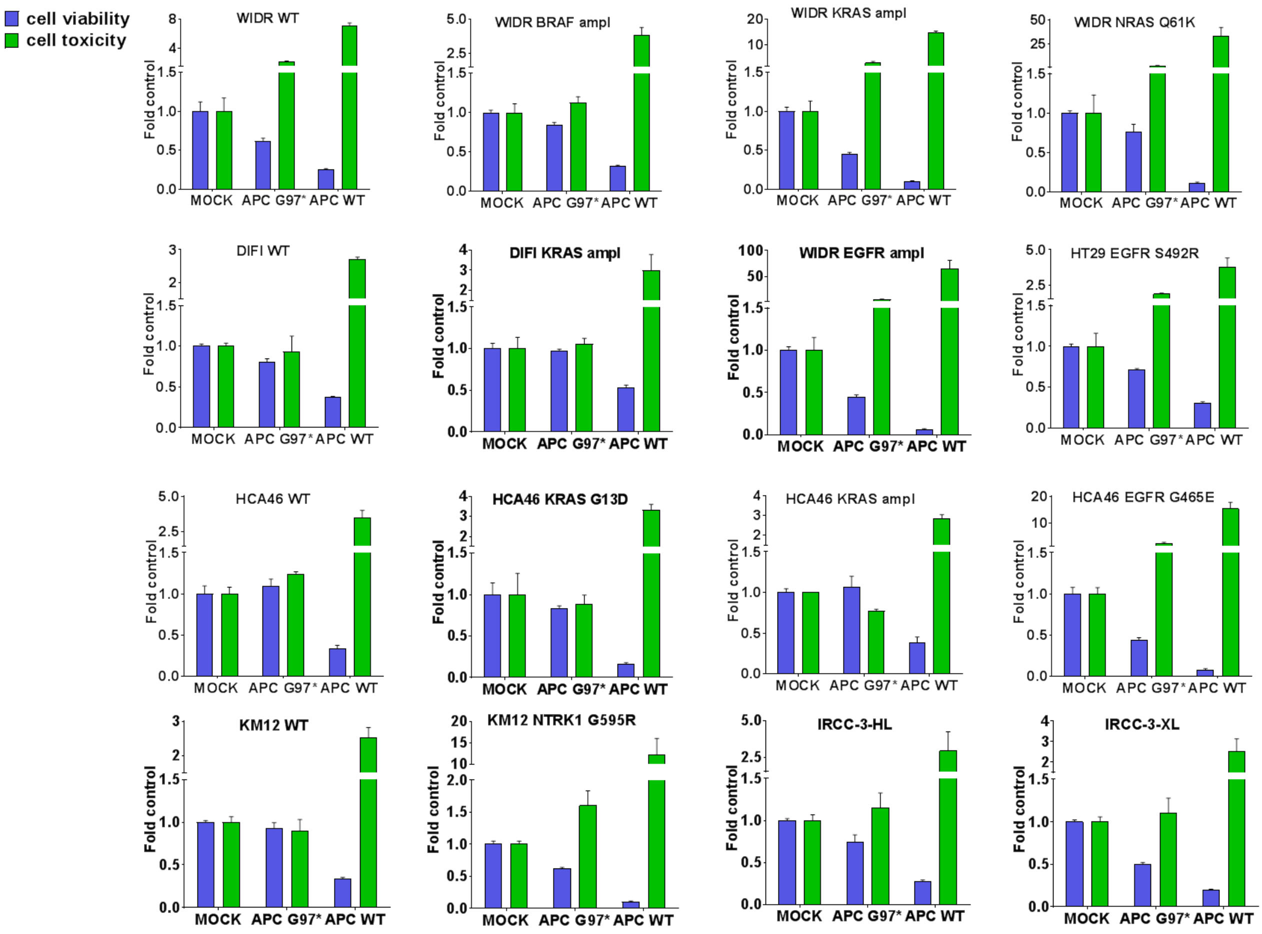

Supplementary Figure 2. *Functional WT APC induces rapid cell death in APC defective CRC cells resistant to MAPK inhibition.* Indicated parental and resistant derivatives CRC cells were transfected with plasmid expressing intact WT APC or an inactive APC version (G97*). Electroporation buffer was used as control (mock). 48h after, cell viability and cell toxicity were assessed by luminescent and fluorescent assays respectively. Results represent means ± SD of three independent wells.

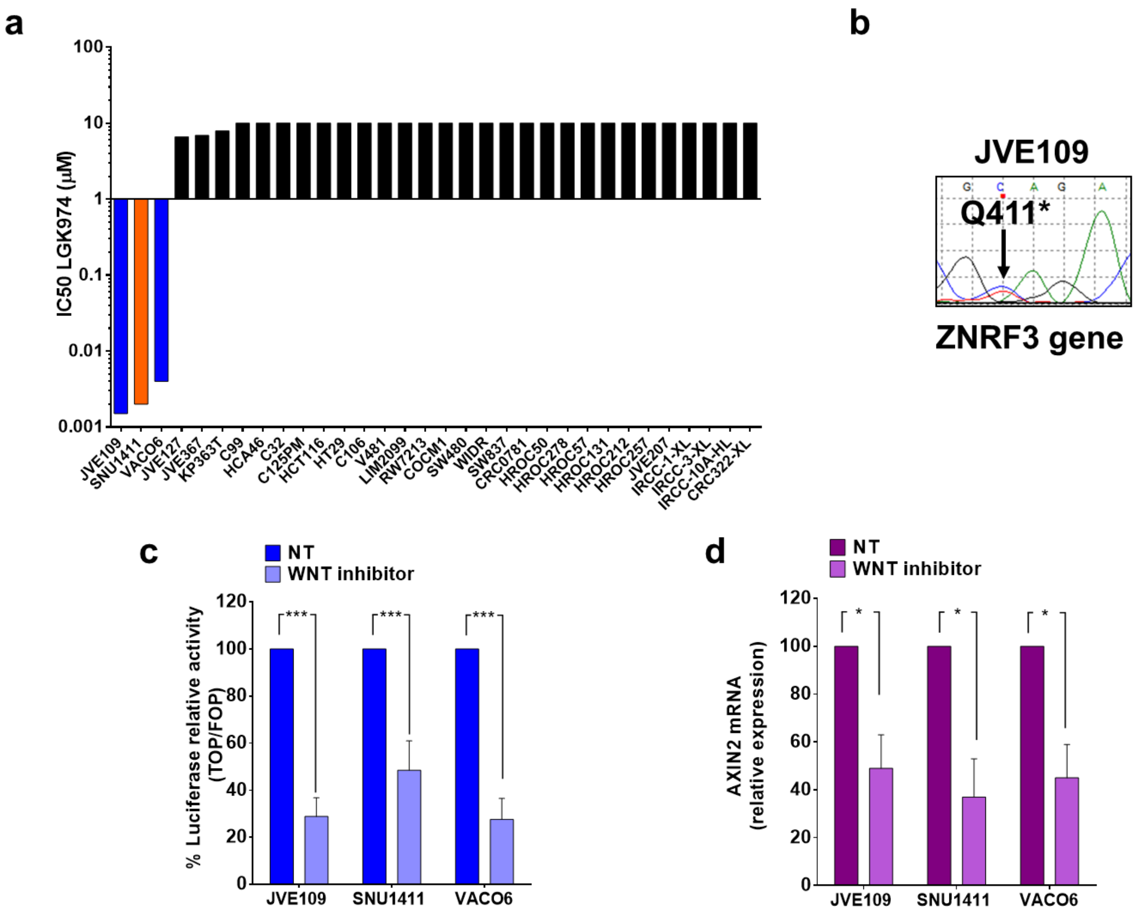

Supplementary Figure 3. *Cell viability screening unveils CRC cells with exquisite sensitivity to WNT pathway inhibition.* (a) The indicated parental CRC cells were treated with increasing concentrations of LGK974 for 5 days. IC50 values reported are the results of at least three independent experiments performed for each cell line. When IC50>10μM, 10 μM is reported. Black bars indicate resistant cells. BRAF V600E (blue bars) and KRAS G12C (red bar) LGK974-sensitive cells are highlighted. (b) *ZNRF3* stop codon mutation was identified and confirmed by Sanger sequence in JVE109 CRC cells. (c) LGK974 treatment dowregulates β-catenin-dependent transcriptional activity of Tcf/LEF luciferase reporter construct in CRC cells harboring genetic alterations in *RSPO3* and *ZNRF3* genes. Results represent means ± SD of at least two independent experiments. ***$p < 0.001$ (Student's t test). (d) Treatment with LGK974 induces a specific downregulation of mRNA level of WNT pathway target gene *AXIN2* in CRC cells with *RSPO3* and *ZNRF3* alterations. Results represent means ± SD of two independent experiments. *$p < 0.05$ (Student's t test).

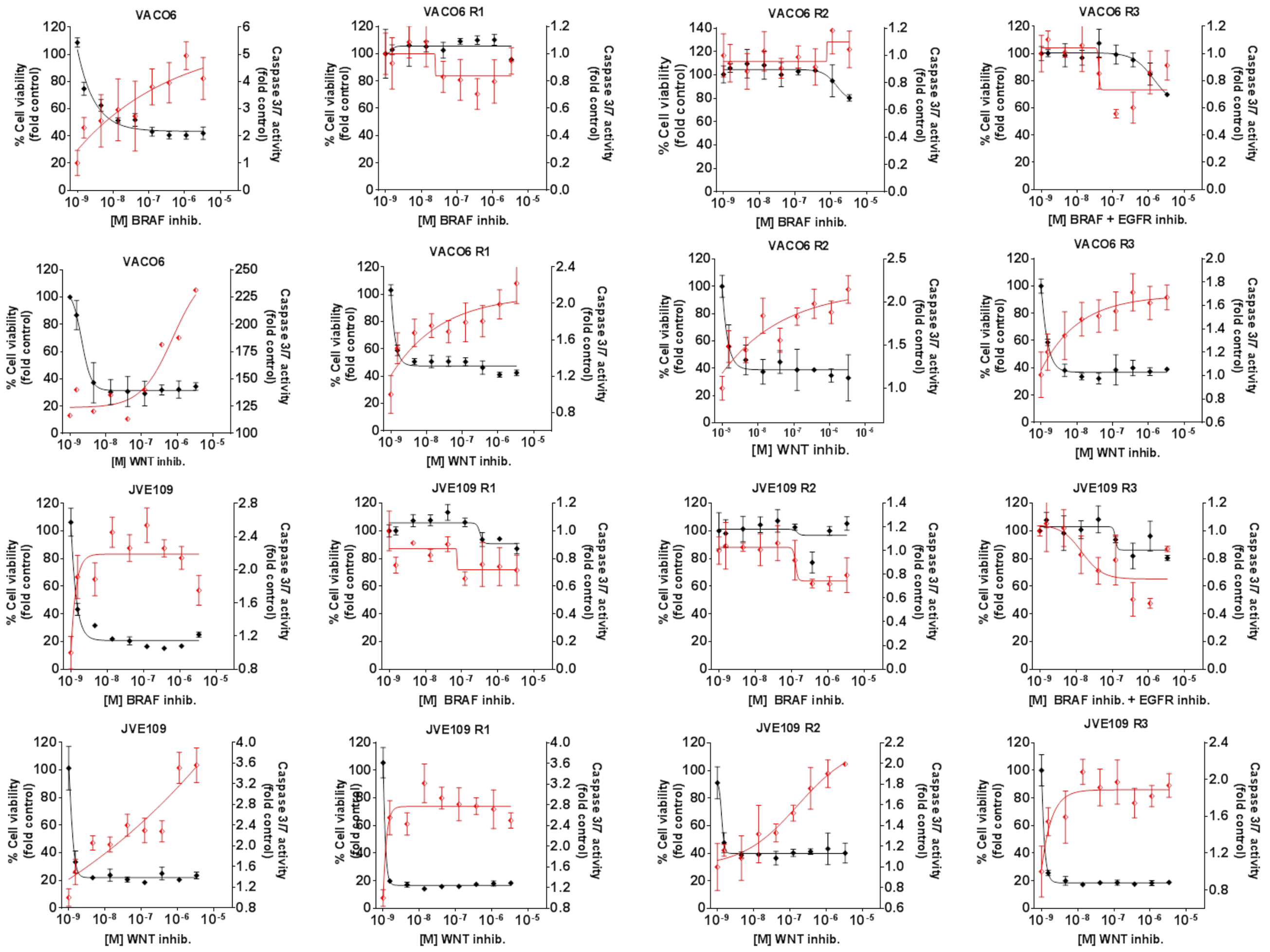

Supplementary Figure 4. *Pharmacological inhibition of trunk WNT pathway leads to apoptosis in drug resistant cells.* CRC parental and resistant derivatives cells were treated with dabrafeninb (BRAF inhib.), dabrafenib + cetuximab (EGFR inhib.) or LGK974 (WNT inhib.). After 5 days, cell viability (black line, left y-axis) and caspase 3/7 activity (red line, right y-axis) were measured. Representative graphs of at least two independent experiments for each condition are reported.

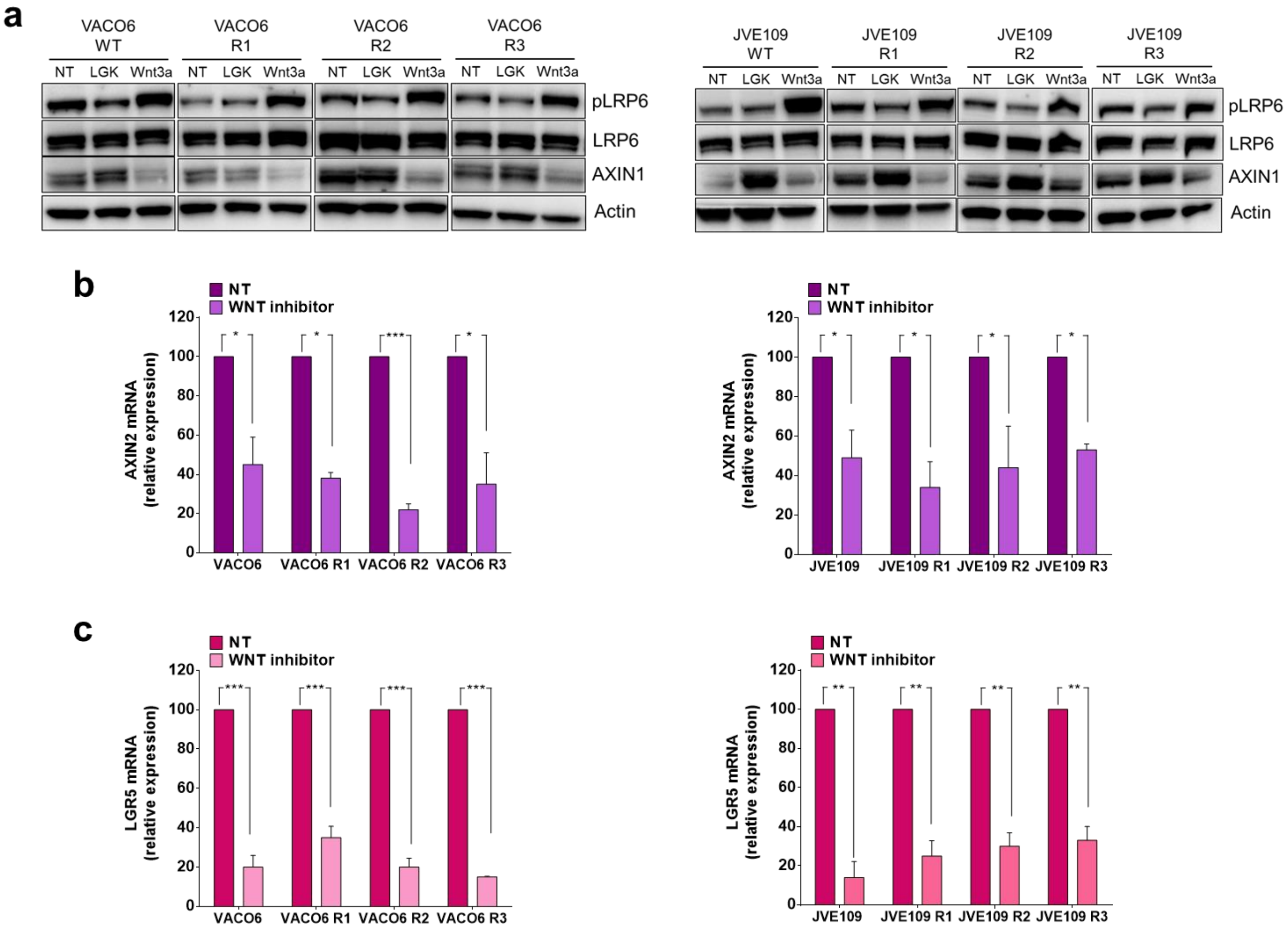

Supplementary Figure 5. *CRC cells with acquired resistance to MAPK inhibitors remain sensitive to WNT pathway modulation.* (a) CRC parental and resistant cells were treated with 1µM LGK974 for 24h or with 100ng/ml WNT3a for 1h. NT indicates untreated cells. Expression levels of WNT pathway genes *AXIN2* (b) and *LGR5* (c) were evaluated in indicated cells treated with 1µM LGK974 (WNT inhibitor) for 24h. Results represent means ± SD of at least two independent experiments. *$p < 0.05$, **$p < 0.01$, ***$p < 0.001$ (Student's t test).

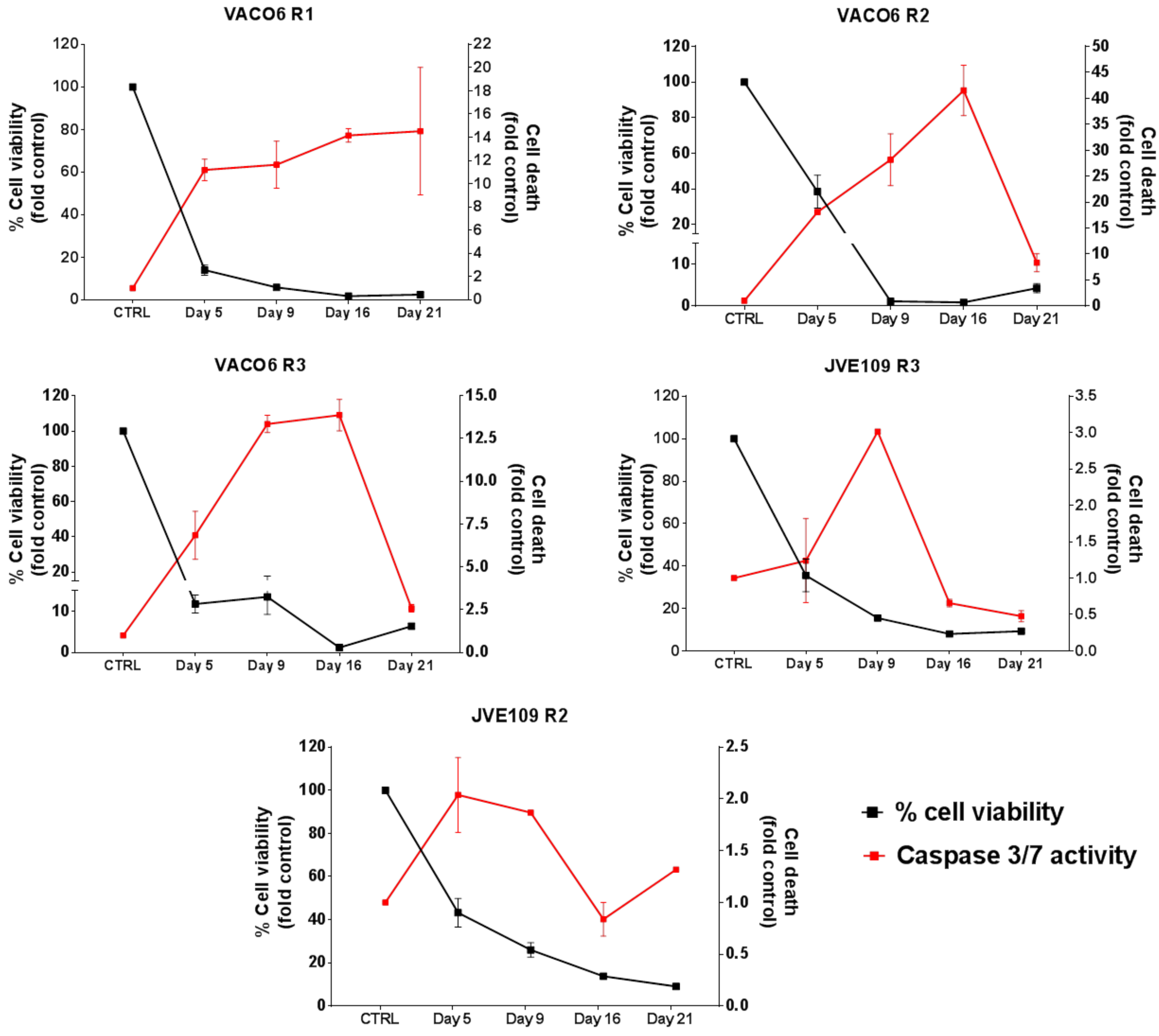

Supplementary Figure 6. *Long-term effect of WNT modulation on CRC resistant cells.* MAPK-resistant cells were seeded in 48wells and treated with 1µM LGK974. Drug treatment was repeated every week. Cell viability (black line, left y-axis) and cell death (caspase 3/7 activity red line, right y-axis) were analyzed at the indicated time points. DMSO treated cells were used as control (CTRL).

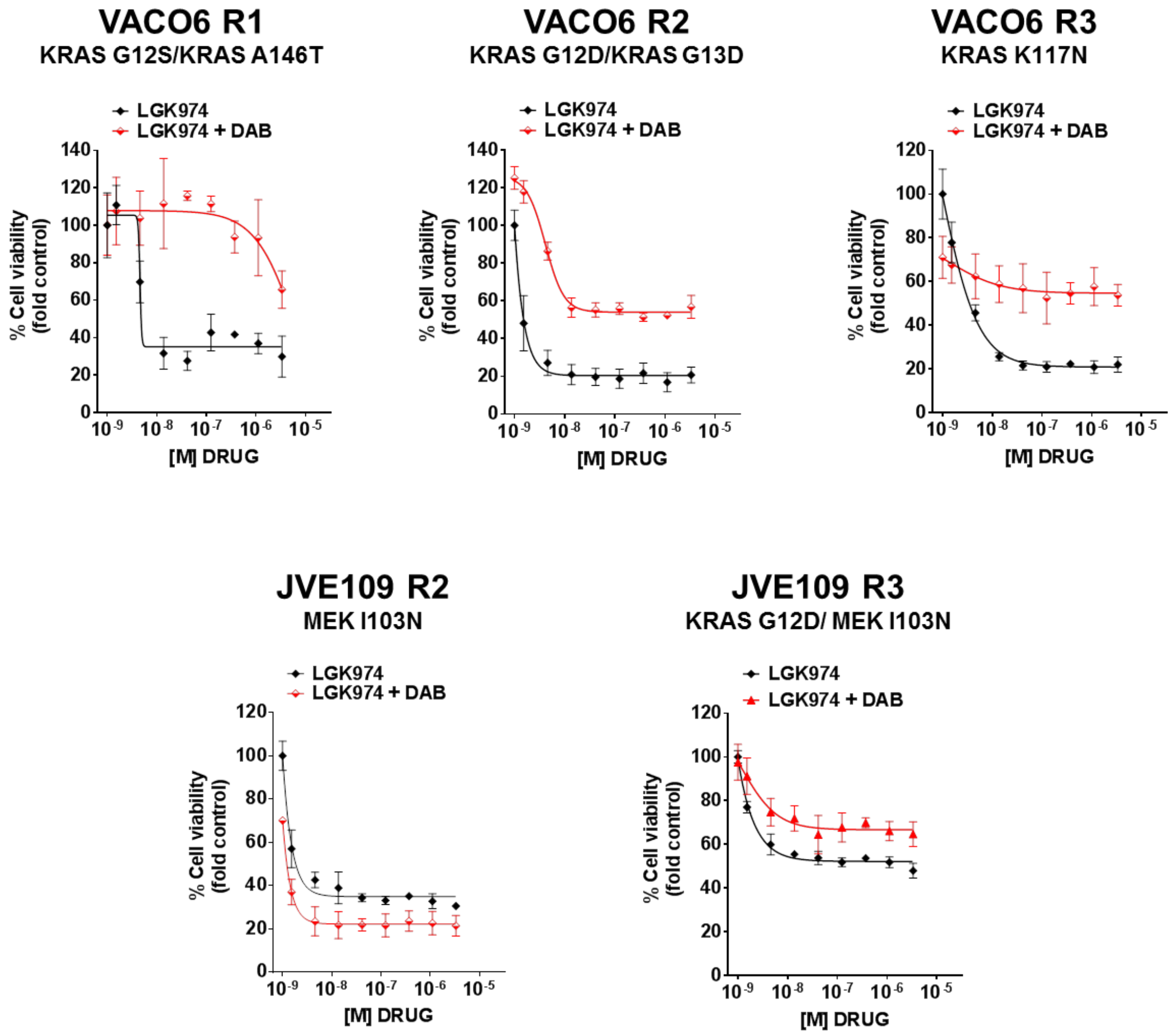

Supplementary Figure 7. *MAPK inhibitor reduces the ability of WNT inhibitor to impair tumor growth of MAPK-resistant CRC cells.* MAPK-resistant CRC cells were treated with LGK974 alone or in combination with MAPK inhibitor dabrafenib (DAB). After 5 days, cell viability was assayed by ATP assay. Mutations known to drive secondary resistance are reported.

CRC cell line	WNT pathway alterations	Sensitivity to target agent(s)	Mechanisms of resistance to target agents
**WIDR**	*APC* p.E1536fs*4/E853*	vemurafenib + selumetinib (R1)	EGFR amplification
		dabrafenib + cetuximab (R2)	KRAS amplification
		vemurafeninb + cetuximab (R3)	NRAS Q61K
		selumetinib + cetuximab (R4)	BRAF V600E amplification
**HT29**	*APC* p. E1536fs*4/E853*	dabrafenib + cetuximab (R1)	EGFR S492R<br>KRAS E63K
**HCA46**	*APC* p.R213*	cetuximab (R1)	KRAS amplification
		cetuximab (R2)	KRAS G13D
		cetuximab (R5)	EGFR G465E
**DIFI**	*APC* p.E425fs*15/E1151*	cetuximab (R2)	KRAS amplification
**KM12**	*APC* p.N1818fs*2/p.G471E	entrectinib (R1)	NTRK1 G595R
**VACO6**	*PTPRK-RSPO3* translocation	dabrafenib (R1)	KRAS G12S/A146T
		dabrafenib (R2)	KRAS G12D/G13D
		dabrafenib + cetuximab (R3)	KRAS K117N
**JVE109**	*PTPRK-RSPO3* translocation	dabrafenib (R1)	KRAS G13D
		dabrafenib (R2)	MEK1 I103N
		dabrafenib + cetuximab (R3)	KRAS G12D<br>MEK1 I103N
**IRCC-3-XL**	*APC* p.R499*/R1450*	cetuximab	KRAS G12D<br>BRAF V600E
**IRCC-3-HL**	*APC* p.R499*/R1450*	cetuximab	KRAS G12D

Supplementary Table 1. *CRC cells resistant to MAPK pathway inhibition*. The table lists all CRC cell lines used in the study. Trunk alterations in WNT pathway, sensitivity to specific targeted agent, or combination of them, and alterations acquired at secondary resistance are indicated.

<table>
<tr><th>CRC cell line</th><th>Resistance to target agent(s)</th><th>Mechanisms of resistance</th><th>Frequency of mutated alleles (%)</th></tr>
<tr><td>HCA46</td><td>cetuximab (R2)</td><td>KRAS p.G13D</td><td>20.4</td></tr>
<tr><td rowspan="2">HT29</td><td rowspan="2">dabrafenib + cetuximab (R1)</td><td>EGFR p.S492R</td><td>13.2</td></tr>
<tr><td>KRAS p.E63K</td><td>20</td></tr>
<tr><td>WIDR</td><td>vemurafeninb + cetuximab (R3)</td><td>NRAS p.Q61K</td><td>34.9</td></tr>
<tr><td>KM12</td><td>entrectinib (R1)</td><td>NTRK1 p.G595R</td><td>45.5</td></tr>
<tr><td rowspan="5">VACO6</td><td rowspan="2">dabrafenib (R1)</td><td>KRAS p.G12S</td><td>11.4</td></tr>
<tr><td>KRAS p.A146T</td><td>13.1</td></tr>
<tr><td rowspan="2">dabrafenib (R2)</td><td>KRAS p.G12D</td><td>27.2</td></tr>
<tr><td>KRAS p.G13D</td><td>1.2</td></tr>
<tr><td>dabrafeninb + cetuximab (R3)</td><td>KRAS p.K117N</td><td>33.2</td></tr>
<tr><td rowspan="4">JVE109</td><td>dabrafenib (R1)</td><td>KRAS p.G13D</td><td>8.7</td></tr>
<tr><td>dabrafenib (R2)</td><td>MAP2K1 p.I103N</td><td>23.5</td></tr>
<tr><td rowspan="2">dabrafeninb + cetuximab (R3)</td><td>KRAS p.G12D</td><td>20.9</td></tr>
<tr><td>MAP2K1 p.I103N</td><td>11.7</td></tr>
</table>

Supplementary Table 2. *Mutational analysis of CRC resistant cells.* The table shows mutations identified in CRC cells resistant to different therapeutic regimen. Variants were identified comparing parental and resistant cells, excluding fractional abundances below 1%; their occurrence in the COSMIC database was required.

Sample	Fraction of mutated clones
**HT29 R1**	12/66 EGFR S492R
	3/7 KRAS E63K
	1/7 MIXED
**JVE109 R3**	10/17 KRAS G12D
	4/17 MAP2K1 I103N
	3/17 MIXED

Supplementary Table 3. *Single cell dilution of CRC cells with secondary resistance to MAPK inhibition.* CRC cells were single cell diluted in 96-wells plate, after 4-5 weeks ddPCR analysis was performed on gDNA extracted from individual clones for mutant alleles previously identified in the population.

Sample	HT29 R1-clones	Fractional abundance of EGFR S492R (%)	Fractional abundance of KRAS E63K (%)
HT29 R1	Clone 1		19.1
	Clone 2	25.6	
	Clone 3	24.6	
	Clone 4	21.5	
	Clone 5		39.2
	Clone 6		2.9
	Clone 7	22.5	0.95
**Sample**	**JVE109 R3-clones**	**Fractional abundance of KRAS G12D (%)**	**Fractional abundance of MAP2K1 I103N (%)**
JVE109 R3	Clone 1	38	
	Clone 2		34
	Clone 3	57	
	Clone 4	41	
	Clone 5	32	
	Clone 6		32
	Clone 7	55	
	Clone 8	71	
	Clone 9		33
	Clone 10	48	
	Clone 11		24
	Clone 12	19	
	Clone 13	54	
	Clone 14	60	
	Clone 15	43	7.6
	Clone 16	36.6	9.2
	Clone 17	0.4	34.3

Supplementary Table 4. *Mutational analysis of CRC resistant cells-derived single clones.* Table lists the relative fractional abundance of mutated alleles detected by ddPCR analysis of single clones derived from indicated CRC resistant cell populations.
